# Histone deacetylase inhibitors up-regulate LL-37 expression independent of toll-like receptor mediated signalling in airway epithelial cells

**DOI:** 10.1186/1476-9255-10-15

**Published:** 2013-04-11

**Authors:** Quan Liu, Juan Liu, Kristina Irene Lisolette Roschmann, Danielle van Egmond, Korneliusz Golebski, Wytske Johanna Fokkens, Dehui Wang, Cornelis Maria van Drunen

**Affiliations:** 1 Department of Otolaryngology, Eye, Ear, Nose and Throat Hospital, Fudan University, Shanghai, 200031, China; 2 Department of Otorhinolaryngology, Academic Medical Center, Amsterdam, the Netherlands; 3 Hannover Medical School, SFB 587, Immune reactions of the lung in infection and allergy, Hannover, Germany; 4 Department of Airway Immunology, Fraunhofer Institute for Toxicology and Experimental Medicine, Hannover, Germany

**Keywords:** Epigenetics, Innate immunity, Histone, Deacetylation, Toll-like receptor, Cathelicidins

## Abstract

HDAC inhibitors have been proposed as anticancer agents. However, their roles in innate genes expression remain not well known. Cathelicidin LL-37 is one of the few human bactericidal peptides, but the regulation of histone acetylation on LL-37 expression in airway epithelium remains largely unknown. Therefore, we investigated the effects of two non-selective HDACi, trichostatin A (TSA) and sodium butyrate (SB), on the expression of the cathelicidin LL-37 in human airway epithelial cells. LL37 in human NCI-H292 airway epithelial cells and the primary cultures of normal nasal epithelial cells(PNEC) in response to HDAC inhibitors with or without poly (I:C) stimulation was assessed using real-time PCR and western blot. In parallel, IL-6 expression was evaluated by ELISA. Our results showed that HDAC inhibitors up-regulated LL-37 gene expression independent of poly (I:C) stimulation in PNEC as well as in NCI-H292 cells. HDAC inhibitors increased LL37 protein expression in NCI-H292 cells but not in PNEC. In addition, HDAC inhibitors significantly inhibited poly (I:C)-induced IL-6 production in both of the epithelial cells. In conclusion, HDAC inhibitors directly up-regulated LL-37 gene expression in human airway epithelial cells.

## Introduction

Recent studies suggest that epigenetics have an important role in regulating innate immunity and that the manifestation and severity of diseases may be influenced by epigenetic factors. Epigenetic modifications play an important role in the regulation of gene expression and a common mechanism in epigenetics is the control of the accessibility of the transcriptional machinery to promoter and enhancer elements in the genome. Histone modification through reversible acetylation is a crucial event in gene transcription regulation [1-3]. The net state of histone acetylation is regulated by the opposing actions of histone acetyltransferases(HATs) and histone deacetylases (HDACs). Small changes in the HAT/HDAC balance could affect transcription of many inflammatory genes, potentially having a profound effect on the initiation and duration of inflammatory responses [4,5]. Yin *et al*[6] reported on bacteria-specific innate immune responses via epigenetic regulation in gingival epithelial cells.

The respiratory epithelium is an important interface with the environment and it is now well accepted that the epithelium is not only just a physical barrier, but plays an active role in innate and adaptive immunity [7]. Antibacterial peptides are an integral part of the epithelial defence barrier that provides immediate protection against infection. Cathelicidins are a family of antimicrobial peptides and LL-37, the only cathelicidin in humans, plays a critical role in the defension of epithelium against the microorganism and is produced by neutrophils, macrophages, and various epithelial cells as well [8].

Increasing evidence suggests that HDAC inhibitors down-regulated the expression of numerous host defense genes including pattern recognition receptors and cytokines. In this study, we wanted to explore the effect of HDAC inhibitors on the expression of LL-37 in airway epithelium in the context of the viral double-stranded RNA-mimic poly(I:C).

## Materials and methods

### Materials

Bronchial Epithelial Basal Medium (BEBM) with Bronchial Epithelial Growth Media(BEGM) SingleQuots were purchased from Lonza Walkersville, Inc. Poly(I:C), TSA, sodium butyrate, L-glutamine, pronase (type XIV protease) and 0.25% trypsin-0.02% EDTA were purchased from Sigma Chemical Co (St Louis, MO, USA). NCI-H292 human airway epithelial cells were purchased from American Type Culture Collection, Manassas, VA, USA and Shanghai Institutes for Biological Sciences (SIBS). Anti-Cathelicidin antibody was purchased from Abcam. Beta-actin antibody and HRP-linked antibody were purchased from Cell Signaling Technology. Fetal bovine serum, penicillin and streptomycin were purchased from HyClone (Logan, UT, USA). RPMI 1640 and trizol medium were purchased from Life Technologies, Inc., Gaitersburg, MD, USA. Cell Counting Kit 8 was purchased from Dojindo (Kumamoto, Japan). First strand cDNA synthesis kit was purchased from Fermentas GmbH(St Leon-Rot, Germany).

#### NCI-H292 human airway epithelial cell culture

NCI-H292 human airway epithelial cells were cultured as reported before [7]. Briefly, NCI-H292 cells were cultured in RPMI 1640 medium supplemented with 1.25 mM of L-glutamine, 100 U/mL of penicillin, 100 μg/mL of streptomycin and 10% (v/v) of fetal bovine serum in six-well plates. Cells were grown in fully humidified air containing 5% of CO2 at 37°C and were sub-cultured weekly.

#### Isolation and culture of human nasal epithelial cells

Primary nasal epithelial cells (PNEC) were isolated from normal middle turbinate that was obtained from patients who underwent endoscopic endonasal surgery in pituitary adenoma patients who had given their written informed consent in accordance with a study protocol approved by the Ethics Committee of Eye and ENT Hospital of Fudan University. Briefly, the normal middle turbinate was digested using 0.2% pronase in culture medium at 37°C for one hour for dissociation of the mucosal epithelial cells. After digestion, the dissociated cells were washed with PBS, followed by the centrifuge(400 g×5 min). The cell pallet was resuspended with culture medium (BEBM supplemented with BEGM SingleQuots) and plated on a 100 mm culture dish at 37°C for 2 hours to remove fibroblasts, myocytes, and endothelial cells. Then the harvested epithelial cells in the supernatant were grown with culture medium in a 5% CO2 incubator at 37°C. After confluence, the cells were detachment with 0.25% trypsin-0.02% EDTA and then the cells were sub-cultured in 6-well tissue culture plates.

#### Preparation for stimulation experiment

After reaching 80% confluence, the NCI-H292 cells were starved overnight in serum-free RPMI 1640 medium, followed by the stimulation in serum-free medium; PNEC cultured in tissue culture plates were starved overnight in BEBM medium (without BEGM SingleQuots), and subsequently stimulated in BEBM medium.

#### Measurement of cell viability

NCI-H292 and primary nasal epithelial cells viability were assessed 24 h after stimulation by incubating cells with Cell Counting Kit 8 according to the manufacturer’s instructions.

#### Enzyme-linked immunosorbent (ELISA) assay

After stimulation, 1 ml of the supernatant in each well was collected; centrifuge (5000 rpm for 5 min) and the cell-free supernatants were stored at −20°C until analysis. The level of IL-6 in supernatant was analyzed using ELISA. A Standard curve was made in each plate with the highest concentration of 20000 pg/ml followed by 2-fold dilution. Each sample was measured at 1, 10, or 100 times dilution.

#### RNA isolation and Real-time PCR

Total RNA for each sample was isolated using trizol according to manufacturer’s protocol. RNA purification was performed using nucleospin RNA II (Machery-Nagel, Germany). RNA concentration was measured using the nanodrop ND-1000 (NanoDrop Technologies Inc., Wilmington, DE, USA). cDNA was synthesized using the MBI Fermentas first strand cDNA synthesis kit. Polymerase chain reaction was performed on Bio-Rad iCycler (Bio-Rad, Veenendaal, the Netherlands). TaqMan® primer (Roche Molecular Systems, Pleasanton, CA, USA) and probe sequences for GAPDH (glyceraldehyde-3-phosphate dehydrogenase) was obtained from Sigma-Aldrich (Haverhill, UK). The sequences for PCR reactions are: GAPDH; sense: 5’-GAA-GGTGAA-GGT-CGG-AGT-C-3^’^,probe:5’-Texas red-CAA-GCT-TCCCGT-TCT-CAG-CC-BHQ2-3^’^, antisense: 5’-GAA-GAT-GGTGAT-GGG-ATT-TC-3^’^. For the other genes, we ordered TaqMangene expression assays from Applied Biosystems (Nieuwerkerka/dIJssel, the Netherlands) with the following IDs: LL37; Hs00189038_m1. TLR3; Hs01551077_m1. Expression changes are presented as ΔΔct, indicating the difference in threshold cycle between active sample and negative control, after correcting for the expression of the housekeeping gene.

#### Quantitative measurement of LL37 protein

Cells were lysed in RIPA. The protein concentration was determined using Protein Assay Solution (Beyotime, China). Equal amount of denaturation proteins were separated on a SDS-PAGE on a 12% glycine-based gel, followed by being transferred to a polyvinylidene difluoride membrane (Millipore, USA), and nonspecific binding sites were blocked. The membrane was incubated with mouse anti-human LL37 antibody (1:1000 dilution, abcam ab87701). The membrane was then incubated with the corresponding horseradish peroxidase labeled secondary goat anti-mouse IgG antibody (1:2500). Immunoreactive proteins were detected with the enhanced chemiluminescence (ECL) western blot detection system (Beyotime, China). b-actin protein was added as the endogenous reference.

#### Statistical analysis

Each set of results shown is representative of at least three separate experiments. Experiments were performed in triplicate and values are shown as the mean ± SD. Statistical significance was determined using the non-parametric Kruskal–Wallis test for variance. When the result was significant, the Mann–Whitney U test was performed for comparisons between groups (SPSS software). All reported P values are 2-sided, and values less than 0.05 were considered to indicate statistical significance.

## Results

### HDAC inhibitors directly induce LL-37 gene expression in NCI-H292 human airway epithelial cells

**Figure 1:**
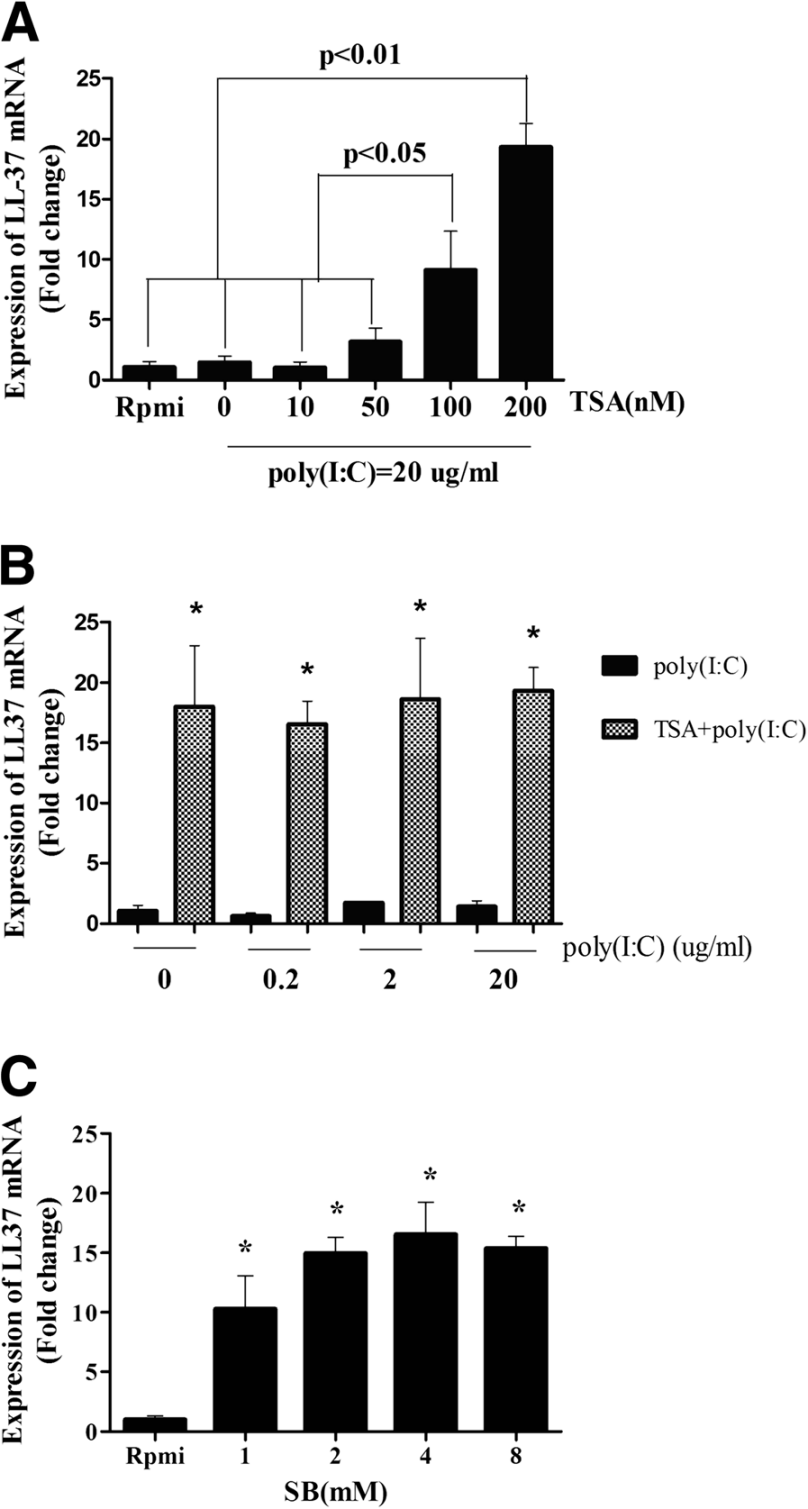
**Real-time polymerase chain reaction analysis of expression of LL-37 in H292 cells in response to poly(I:C) with or without TSA.** (**A**): The mRNA expression of LL-37 at different concentration of TSA in response to the poly(I:C). (**B**): Cells were stimulated with different concentration of poly(I:C) for 24 h in the presence or absence of TSA(200 nM). (**C**): The effect of SB on the LL37 gene expression. *p<0.05 vs control. Values represent the mean±SD of three independent experiments.

Antibacterial peptides are an integral part of the epithelial defence barrier that provides immediate protection against infection. To characterize the role of epigenetics in the expression of human cathelicidin, we assessed LL-37 expression with or without of HDAC inhibitors. Compared to the control group, poly(I:C) by itself slightly increased LL-37 expression. Importantly, expression of LL-37 in the presence of poly(I:C) is further increased to 19-fold (p < 0.01) at increasing concentrations of TSA (Figure 1A). This increase expression induced by TSA seems a direct effect of TSA as it is also observed in the absence of poly(I:C) as seen in Figure 1B.

To confirm the findings obtained with TSA, we tested the effect of other HDAC inhibitor, SB. Like TSA, SB used at concentrations (1 mM, 2 mM, 4 mM and 8 mM) dose dependently increased LL37 expression in the NCI-H292 cell (Figure 1C).

Our results indicate that TSA(200 nM) or SB(4 mM) stimulation for 24 h could effectively up-regulate LL37 gene expression, so, we use TSA(200 nM) or SB(4 mM) through our following experiment.

### HDAC inhibitors induce cathelicidin LL-37 gene expression in human primary nasal epithelial cell

**Figure 2:**
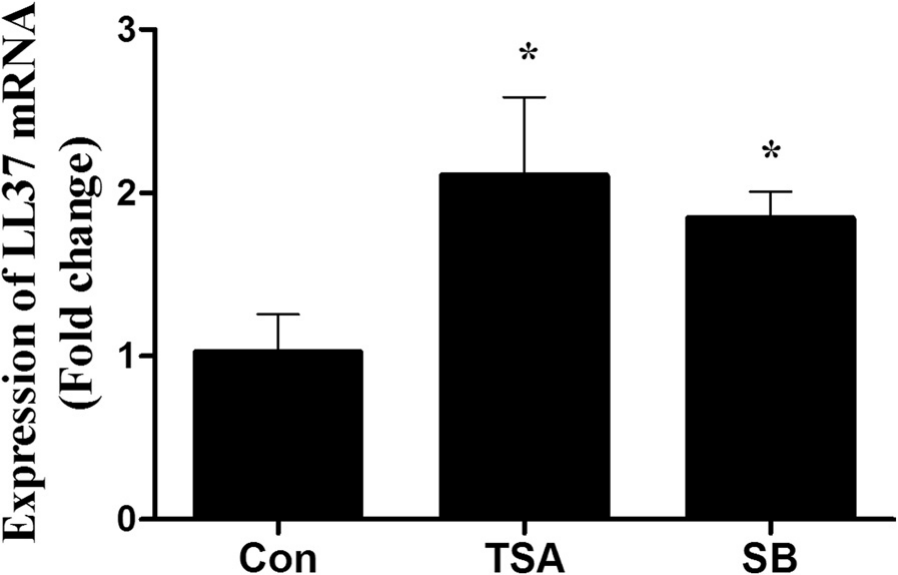
**The effect of HDAC inhibitors ( TSA,200 nM; SB,4 Mm) on the LL37 gene expression in the primary nasal epithelial cell.** *p<0.05 vs control. Values represent the mean±SD of three independent experiments.

The sinonasal tract lined by respiratory epithelium plays an important role in airway immunity. The only human cathelicidin LL37 first identified in neutrophils was shown to be expressed in surface epithelial cells of the conducting airways [9]. To verify whether HDAC inhibitors induce LL37 gene expression in upper airway epithelial cells, we cultured the human nasal epithelial cells and performed the stimulation experiments in the primary cells. Our results demonstrated that the HDAC inhibitors had a similar effect on the LL37 mRNA expression as they did in H292 cells (Figure 2).

### HDAC inhibitors up-regulate LL37 protein expression in NCI-H292 human airway epithelial cells but not in primary nasal epithelial cells

**Figure 3:**
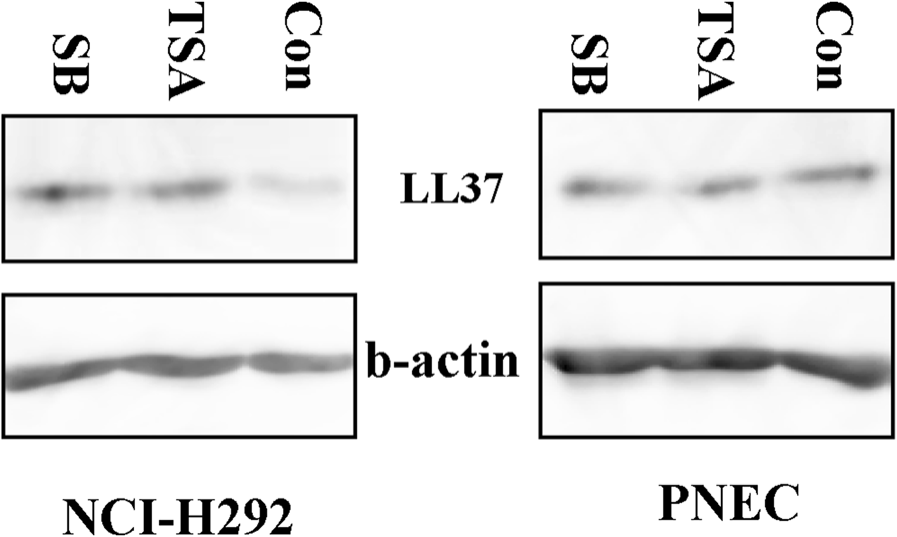
**The LL37 protein expression induced by HDAC inhibitors in the NCI-H292 cells and the primary nasal epithelial cells.** Whole cell lysates prepared from the NCI-H292 cells and PNEC 24 h after treatment with TSA(200 nM) and SB(4 mM). Data shown are from a single representative experiment. These experiments were repeated at least twice to confirm reproducibility.

To analyse the effect of HDAC inhibitors on the LL37 protein expression in the epithelial cells, we treated the NCI-H292 cells and human primary nasal epithelial cells with HDAC inhibitors for 24 hours, followed by the extract of cell total protein and western blot analysis. Our results indicated that the two HDAC inhibitors induced LL37 protein expression in the NCI-H292 cells. However, no significant difference of LL37 protein expression was found in the primary cells (Figure 3).

### HDAC inhibitors suppress IL-6 production after poly(I:C) stimulation

**Figure 4:**
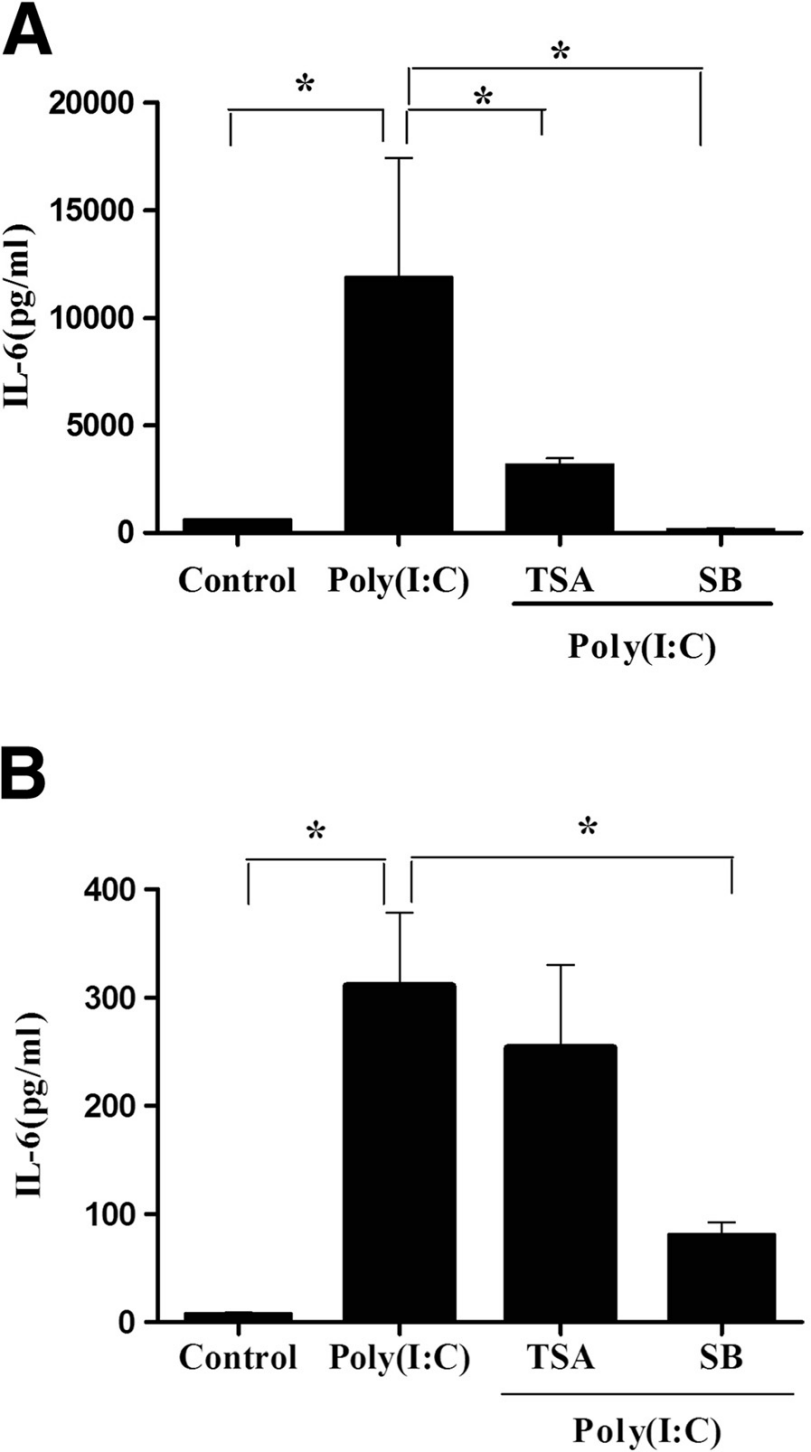
**ELISA analysis of IL-6 expression in the airway epithelial cells.** The effect of HDAC inhibitors on IL-6 production in the NCI-H292 cells (**A**) and the primary nasal epithelial cells (**B**) in response to poly(I:C) stimulation. Cells were pretreated respectively with TSA (200 nM) or SB(4 mM) for 2 h prior to the poly(I:C) (20 ug/ml) stimulation for 24 h. IL-6 production in cell supernatant was analyzed using ELISA. *p<0.05 vs control. Values represent the mean±SD of three independent experiments.

TSA was recently reported to inhibit IL-6 production from monocytes and macrophages [10]. To determine if HDAC inhibitors could also suppress IL-6 production in the airway epithelium, we treated the H292 cells and primary nasal epithelial cells with HDAC inhibitors for 2 h prior to poly(I:C) stimulation. In our experiment, poly(I:C) stimulation for 24 h significantly increased IL-6 protein expression level in both of the airway epithelial cells. Interestingly, we found that pre-incubation with HDAC inhibitors inhibited the IL-6 protein expression in H292 cells (Figure 4A). In the primary nasal epithelial cells, only SB significantly induced IL-6 expression (Figure 4B).

### The effect of HDAC inhibitors on TLR3 expression in airway epithelial cells

The inhibition of HDAC inhibitors on poly(I:C) induced expression of IL-6 we observed in the previous experiment could be mediated at many different levels. To explore whether some of the inhibitory effect could be upstream of the IL-6 genes we determined TLR3 expression levels as a measure of different HDAC inhibitors concentrations. Our results showed that poly(I:C) stimulation without TSA or SB increased the TLR3 expression by more than one and a half times, and in the presence of different concentrations of HDAC inhibitors, the induced expression of TLR3 gene expression was not seen significantly alternative expression(data not shown), indicating that the inhibition of HDAC inhibitors on poly(I:C) induced expression of IL-6 was not due to TLR3 expression levels.

In this study, cell viability after the stimulation was assessed by the Cell Counting Kit 8. Our data showed that the stimulation with various concentration of poly(I:C), TSA or SB had a minimal effect on cell viability.

## Discussion

In the present study, we have shown a complex interplay between epigenetics and aspects of the innate immune reaction in airway epithelial cells. HDAC inhibitors on one hand inhibit poly(I:C)-induced expression of IL-6, while on the other hand they directly induces LL-37 expression in NCI-H292 human airway epithelial cells. In the primary nasal epithelial cells, we found that only SB inhibited poly(I:C)-induced expression of IL-6 and that both TSA and SB could induce LL37 gene, not protein, expression. Our results indicate that epigenetic regulation plays an important, yet complicated, role in the regulation of innate immunity in airway epithelial cells.

All these observations of inhibition under unstimulated or stimulated conditions seem contrary to what one would expect for the action of an inhibitor of deacetylases. As this inhibition would lead to higher levels of (local) histone acetylation one might expect increased levels of gene activity. In our experiments only the expression of LL-37 seems to follow the expected paradigm. However, TSA and SB might act indirectly on a target gene by affecting the expression of some negative regulator only, or in combination with a positive effect on either the target gene itself or some positive regulator.

Epithelium derived antimicrobial peptide LL-37 is an important component of host defense at mucosal surfaces and exposure to TLR3 agonist is indeed able to up-regulate the expression of LL-37 in primary human corneal epithelial cells [11], just like it was in the airway epithelial cells. However, the positive effects of TSA and SB were much stronger than that of the TLR3 activator and, moreover, this activation does not require the presence of the TLR3 agonist. The positive effect of TSA and SB on the gene expression of LL-37 in airway epithelium is consistent with previous study reported by Schauber *et al*. that histone-deacetylase inhibitors (butyrate) induce the cathelicidin LL-37 in gastrointestinal cells. And they further demonstrated that butyrate induced expression of LL-37 was mediated by MEK-ERK signalling pathway [12]. The different expression of LL37 protein in primary nasal epithelial cells and NCI-H292 cells needs further research.

What is the mechanism underlying HDAC inhibitors induced LL37 expression? Emerging evidence indicates that HDAC inhibitors play an important role in the modulation of core histone (H3 and H4) and non-histone proteins. Butyrate and TSA were reported to induce LL37 expression via acetylation of the non-histone protein HMG-N2 and the histone protein H4 in HT-29 colon, 23132/87 gastric and HepG2 hepatoma cells [12]. LL-37 gene had potential binding sites for several transcription factors, including NF-kB and activator protein-1 [13]. Kuwano *et al*[14] reported sodium butyrate induced histone acetylation of the cathelicidin promoter using the chromatin immunoprecipitation (ChIP) in the human lung epithelial cell line. They also indicated that activator protein-1 plays an important role in the regulation of sodium butyrate-induced transactivation of cathelicidin promoter. In the present study, our results revealed that TSA and SB induced LL37 expression both in gene and protein levels in NCI-H292 cells, which is consistent with the previous reports. Unlike the previously reported effect of HDAC inhibitors on the LL37 expression, Schauber *et al.* indicated that HDAC inhibitors(TSA and butyrate) alone did not change cathelicidin transcript abundance in keratinocytes. They demonstrated that HDAC inhibition significantly amplify cathelicidin expression in keratinocytes in the presence of 1,25-Dihydroxyvitamin D3 [15]. So, we speculate that acetylation of cathelicidin promoter play an important role in LL37 expression. Our results in the nasal epithelial cells indicated that HDAC inhibitors could induce LL37 gene expression, but not the LL37 protein. These observations show that the nature of a response to histone acetylation will be cell-type and gene-specific.

The airway epithelium itself is responsible for the synthesis and release of cytokines that cause the selective recruitment, retention, and accumulation of various inflammatory cells [16,17]. Target cells of the epithelium can respond to a variety of inflammatory mediators and cytokines. IL-6 is a multifunctional cytokine that regulates the immune response, the acute phase response and inflammation. IL-6 is involved in the pathogenesis of lung diseases such as asthma and chronic obstructive pulmonary disease [18]. Our results demonstrated a suppressive effect on IL-6 expression in TSA-exposed airway epithelial cells. These observation are in line with those of Grabiec*et al*[15] that also reported that TSA significantly reduced the production of IL-6 after exposure to multiple stimuli, including poly(I:C), in fibroblast-like synoviocyte and macrophages. Although this group did not investigate TLR3 expression they indicated that the inhibitory effect of TSA was a consequence of accelerated mRNA decay. Our observation of a direct effect of TSA on TLR3 is supported by similar observations in human microglia and astrocytes in their response to poly(I:C) [19].

In addition to the expression of individual genes, the global character of the action of TSA is probably also the reason for its ability to suppress cell growth by inducing cell cycle arrest and to promote differentiation of normal and transformed cells [20]. Increasing evidence suggests that HDAC inhibitors are indeed potent anti-inflammatory and immunomodulatory agents [21,22].

In summary, our results indicate that regulation of histone acetylation and chromatin remodelling plays a complex role in innate immune responses in airway epithelium.

## Competing interest

All authors declare that they have no competing interest.

## Authors’ contributions

QL, JL, KILR, WJF, CMvD and DHW contributed to the design and coordination of this study. QL, JL, DvE and KG carried out all of the studies. QL, JL, CMvD and DHW contributed to the analysis of all the data. QL, JL and DHW drafted the article. WJF, CMvD and DHW revised the manuscript. All authors read and approved the final manuscript.
